# High-resolution climate models improve simulation of monsoon rainfall changes in the Ganga–Brahmaputra-Meghna basin

**DOI:** 10.1007/s00382-025-07716-6

**Published:** 2025-06-06

**Authors:** Haider Ali, Hayley J. Fowler, Andrew G. Turner

**Affiliations:** 1 School of Engineering, Newcastle University, Newcastle Upon Tyne, UK; 2 Tyndall Centre for Climate Change Research, Newcastle University, Newcastle Upon Tyne, UK; 3 National Centre for Atmospheric Science, University of Reading, Reading, UK; 4 Department of Meteorology, University of Reading, Reading, UK

## Abstract

This study investigates the impact of model resolution on simulating South Asian monsoon rainfall, focusing on the Ganges–Brahmaputra-Meghna (GBM) basin. By comparing high- and low-resolution versions of four CMIP6 HighResMIP model families against reference datasets (MSWEP and ERA5), we emphasize the advantages of high-resolution models in accurately simulating key monsoon characteristics, including annual rainfall, timing, intensity, and duration. Our results show that the high-resolution models align more closely with observed data, outperforming their low-resolution counterparts. Between 1979 and 2014, the high-resolution model ensemble (HR-models) captures key shifts in monsoon timing, such as delayed onset and withdrawal, leading to a slight increase in monsoon duration. In contrast, the low-resolution ensemble (LR-models) showed more pronounced delays in onset. The observational datasets, MSWEP and ERA5, indicate earlier (7 ± 3 days) and later (3 ± 1.2 days) onsets, respectively, with both showing delays in withdrawal, indicating extended monsoon duration. Notably, the increase in monsoon duration is more pronounced in MSWEP observations than in the model simulations, particularly for LR-models. Regarding rainfall trends, the HR-models more accurately reflect observed changes in both total rainfall and extreme rainfall from 1979–2014 compared to LR-models. Future projections (2015–2050) indicate further delays in monsoon onset, with HR-models projecting larger increases in total rainfall and extreme events (up to 4.5%/decade for the 95th percentile of rainfall) compared to LR-models, which show smaller increases and higher variability in total and extreme rainfall. These findings highlight the critical role of model resolution in improving the accuracy of monsoon simulations, with HR models offering more reliable simulations of historical monsoon behaviour and therefore likely more robust projections of future monsoon behavior. These are essential for informed water management and agricultural decision-making over the complex topography of the GBM basin.

**Supplementary Information:**

The online version contains supplementary material available at 10.1007/s00382-025-07716-6.

## Introduction

The monsoon system plays a critical role in the hydrological cycle and particularly impacts precipitation in South Asia (Serreze and Barry 2010). Among the most densely populated agricultural basins globally, the Ganges–Brahmaputra-Meghna (GBM) basin relies heavily on South Asian monsoon rainfall (Ali et al. 2023; Azad et al. 2022). Monsoon rainfall profoundly affects agricultural production, environmental sustainability, and water resource management in the basin (Rahman et al. 2017; Gadgil and Gadgil, 2003). Variations in monsoon timing, intensity, and duration significantly impact agricultural productivity, food security, hydroelectric production, forest vegetation, water resources, and regional ecology (Turner and Annamalai 2012; Jain et al. 2013). Therefore, a detailed analysis of monsoon rainfall characteristics, including timing, total and extreme rainfall amounts, and trends, is essential for understanding the implications for water resources and the economy in this basin (Mandal et al. 2021; Rahman et al. 2017).

Extensive research has focused on the timing of the South Asian monsoon, considering both regional and large-scale patterns and trends (Azad et al. 2022; Bombardi et al. 2020; Misra et al. 2018; Montes et al. 2021). These studies have utilized different criteria and atmospheric variables to analyze variations in monsoon timing and associated forcing mechanisms, including the assessments of long-term trends. Monsoon onset and retreat are influenced by a combination of local and regional factors, leading to multiple proposed explanations (Wang et al. 2017). In addition to fundamental large-scale factors such as continental heating and meridional wind shifts, mechanisms such as intraseasonal oscillations and forcing from convection over the oceans, especially in the Bay of Bengal, play a significant role (Fasullo and Webster 2003; Karmakar and Misra, 2019). Furthermore, sea surface temperature anomalies in the Indian and Pacific Oceans, along with El Niño/La Niña events, contribute to variations in monsoon onset timing, impacting the GBM basin (Sun et al. 2017; Xavier et al. 2007).

Global climate models (GCMs) help us to understand the changes in monsoon rainfall by attempting to reproduce its past changes and make projections of its future (Zhu et al., 2020). However, simulating monsoon precipitation at regional scales using these models presents challenges, primarily due to limitations in their resolution and their inability to fully represent many of the smaller-scale processes that govern regional precipitation (Haarsma et al. 2016). This results in systematic and persistent biases when compared to observations, raising concerns about model reliability and reducing our confidence in future climate projections (Roberts et al. 2019).

To enhance the representation of regional precipitation, downscaling techniques are often employed. These methods bridge the gap between the coarse resolution of GCMs and the fine-scale data required for regional impact assessments (Xin et al. 2021). Regional Climate Models (RCMs), which downscale Earth System Model (ESM) outputs, are particularly useful in capturing regional topography, land–ocean contrasts, and the associated climate processes (Avila-Diaz et al. 2023; Ban et al. 2021). While RCMs offer greater spatial detail, they can introduce uncertainties, such as boundary condition closure issues (Giorgi 2006; Ambrizzi et al. 2019). To address this, high-resolution ESMs are being developed, aiming to provide comprehensive regional and global climate data while incorporating more climate processes compared to RCMs (Demory et al. 2020). Some of the support for this idea comes from previous comparisons of different climate models in projects like the Coupled Model Intercomparison Project (CMIP) (Roberts et al. 2019; Meehl et al. 2007; Taylor et al. 2012).

While the effectiveness of downscaling at improving climate projections for the Indian summer monsoon remains uncertain, high-resolution models have shown improvements in precipitation projections in complex orographic regions, where such improvements are crucial. The IPCC AR6 Chapter 10 (Doblas-Reyes et al. 2021) provides strong evidence of the value of high-resolution simulations in these areas. Johnson et al. (2016) highlighted that higher-resolution models improve the representation of precipitation processes over regions like the Western Ghats mountains, although challenges such as the dry bias over South Asia and the wet bias over the Indian Ocean persist. Yet, Bock et al. (2020) argue that CMIP6 models show no significant improvement over CMIP5 or CMIP3 models regarding annual mean rainfall biases in the tropics, and the High Resolution Model Intercomparison Project (HighResMIP) models do not significantly reduce the overall bias at the large scale compared to lower-resolution models. Despite these limitations, we contend that high-resolution models offer improved representation of precipitation processes in areas with complex topography, such as the GBM basin, making them valuable tools for conducting such studies.

The HighResMIP, endorsed by CMIP6, introduces a novel multi-model approach to systematically explore the effects of horizontal resolution for the first time (Haarsma et al. 2016). These simulations vary in resolution from typical CMIP6 values (~ 250 km in the atmosphere and 100 km in the ocean) to significantly higher resolutions (25 km in the atmosphere and 8 to 25 km in the ocean). In HighResMIP, each model performs parallel experiments at both high and low resolutions, using identical parametrizations and tunings. To assess the resolution improvement, the high-resolution and low-resolution experiments are compared within the context of the same model. There have already been some relevant analyses to show the utility of HighResMIP models in simulating characteristics of the South Asian monsoon. For instance, Fahad et al. (2022) found that the low-resolution simulations from HighResMIP show poor spatial variability of precipitation and a dry bias across Bangladesh; however, the high-resolution coupled simulations have a better representation of topography, which improves the simulation of moisture convergence at the foothills of the Himalaya and reduces precipitation biases.

Here, we aim to assess the impact of model resolution on monsoon simulations by conducting a comparative analysis of high- and low-resolution experiments within the same model framework. Each model performs parallel simulations at both resolutions, using identical parameterizations and tunings to isolate the effect of resolution on monsoon characteristics. We look at observed and projected changes in the timing (onset, withdrawal, and duration) and strength (total and extreme) of monsoon rainfall in the GBM basin, using both high- and low-resolution models from HighResMIP and reference reanalysis datasets. The key science questions addressed in this study are:

- How do high-resolution and low-resolution versions of the same model family differ in simulating monsoon rainfall in the GBM?
- How accurately do HighResMIP models simulate the timing and strength of monsoon rainfall compared to observed reference datasets, and how does model resolution impact these simulations?
- What are the observed and projected changes in the timing and strength of monsoon rainfall in the GBM basin, and how do these changes vary between high- and low-resolution models?

In Sect. 2, we describe the study region, data, and the definitions of the rainfall indices used in this study. Section 3 presents our results, while Sect. 4 concludes our findings.

## Data & methods

### Study region

**Fig. 1:**
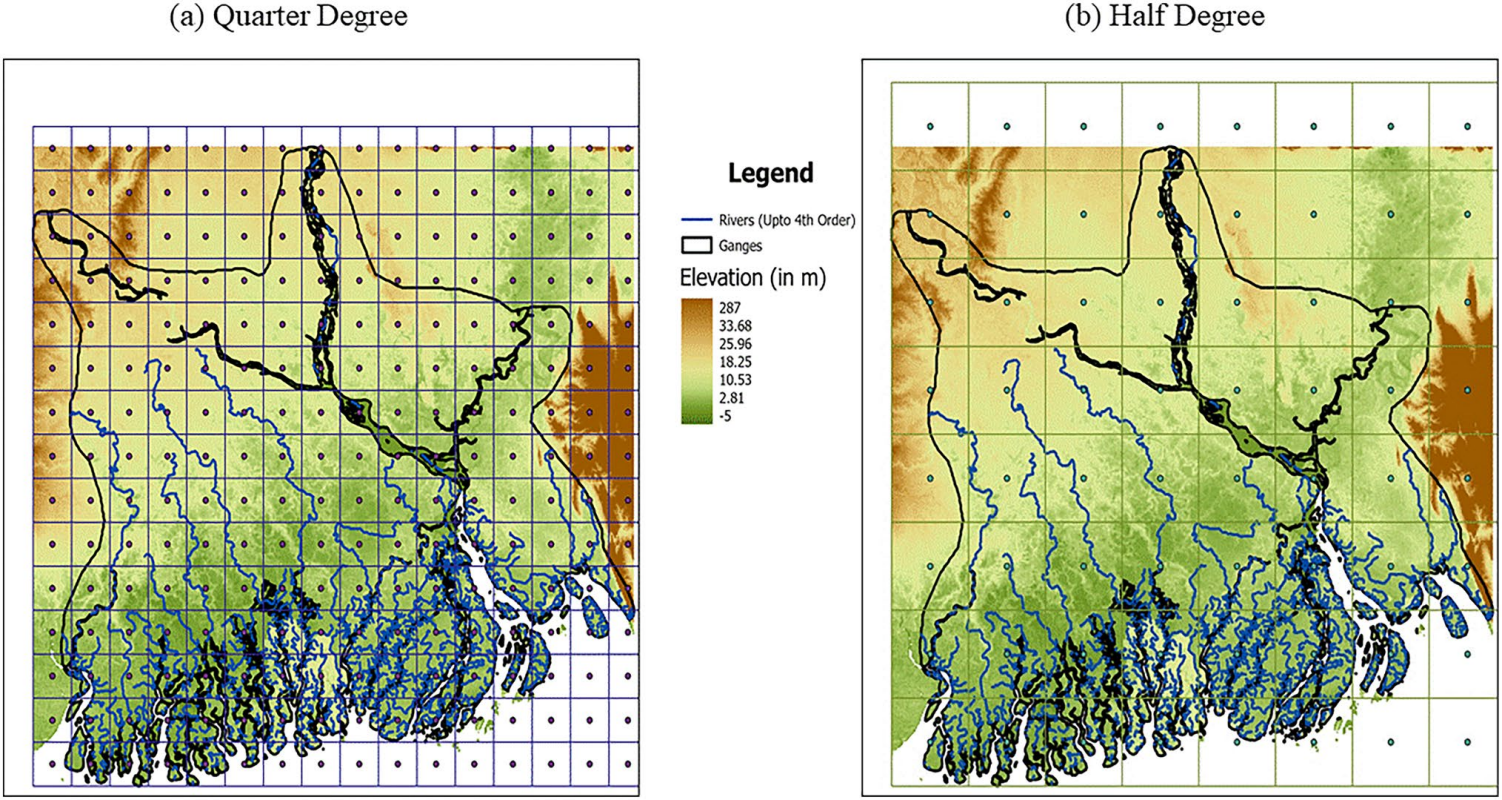
Boundary of the GBM basin showing elevation differences and river networks up to the 4th order. Panels **a** and **b** display the region with grid spacing of 0.25 and 0.5 degrees, respectively

Our study is focused on the Ganges–Brahmaputra-Meghna (the GBM hereafter) basin. The GBM is a river basin located between latitudes 21°25′N to 25°50′N and longitudes 87°75′E to 91°75′E, covering Bangladesh and parts of eastern India. With a catchment area of approximately 1.72 million km^2^, the basin is home to around 630 million people, making it one of the most densely populated regions in the world (Sharma et al. 2021). The GBM is a complex river system characterized by diverse range of topographical and morphological features (Mirza 2002), an intricate river network and varied elevation (from 1 m a.s.l. in the South to 33 m in the North). Figure 1 highlights the complex orography of the basin, emphasizing the need for high-resolution models to accurately capture local-scale processes that are crucial for improving precipitation estimates, which can directly benefit the large population living in the region.

To delineate these basins we used boundaries from the HydroSHEDS website through the link https://hydrosheds.org/downloads.

### Climate reference datasets

Reference climate datasets are essential for assessing the performance of HighResMIP models in simulating monsoon rainfall characteristics and for validating model outputs against observed precipitation patterns. In this study, we utilize two primary reference datasets: the European Centre for Medium-Range Weather Forecasts (ECMWF) Reanalysis v5 (ERA5; Hersbach et al. 2020) and the Multi-source Weighted-Ensemble Precipitation (MSWEP; Beck et al. 2017). The use of these two datasets allows for a more thorough comparison and validation of model outputs, ensuring a comprehensive evaluation of model performance and ultimately strengthening the reliability of the study's conclusions.

ERA5 is a global atmospheric reanalysis product created by ECMWF using the 4D-Var data assimilation techniques in cycle 41r2 (Karl and Michela, 2019). Precipitation in ERA5 is obtained from a combination of data analysis and forecasting and consists of two surface-level parameters: rainfall and snow. Large-scale precipitation in ERA5 is produced by the cloud scheme, while convective precipitation is derived from the convection scheme. ERA5 data are available from 1950 to the present with a temporal output resolution of 1 h and a horizontal 0.25-degree spatial resolution (Hersbach et al. 2020). Mahto and Mishra (2019) found that ERA5 outperforms other reanalysis products (MERRA2, CFSR, ERA-Interim & JRA-55) for monsoon precipitation across India, however, uncertainties remain in tropical regions due to the limited observational data available for the evaluation (Ali et al. 2021a, b).

MSWEP is a new fully-global historic precipitation dataset covering the period from 1979 to 2020. It offers a spatial resolution of 0.25° and a temporal resolution of 3 h. The long-term mean background of MSWEP is derived from the CHPclim dataset and is supplemented with more accurate regional datasets where available (Beck et al. 2017). MSWEP takes advantage of two gauge datasets (CPC Unified and GPCC), three satellite products (CMORPH, GSMaP-MVK, and TMPA 3B42RT), and two reanalyses (ERA-Interim and JRA-55) to provide reliable precipitation estimates globally. Therefore, MSWEP is not strictly a reanalysis dataset. Previously, Ali et al. (2019) used MSWEP to study multiday flooding events in the Indian subcontinent, demonstrating its reliability in capturing extreme rainfall, which justifies its use in this study.. More details about the MSWEP dataset can be found at http://www.gloh2o.org/.

### Model simulations and projections

The HighResMIP experiments, spanning from 1950 to 2050, are categorized into three tiers: atmosphere-only (Tier 1), coupled atmosphere–ocean (Tier 2) and forced-atmosphere (Tier 3) with potential extension to 2100, alongside additional targeted experiments. Tier 1 experiments, named HighResSST-present, involve historically-forced atmosphere runs from 1950 to 2014 (ForcedAtmos) using the HadISST2.2.0.0 1/4 degree sea-surface temperature (SST) and sea-ice forcing dataset, with fixed land use following the HighResMIP protocol (Haarsma et al. 2016). Tier 2 consists of simulations: a) conducted over 100 years using forcing conditions from the 1950s – “control-1950”; b) spanning from 1950 to 2014 using historical forcing conditions including greenhouse gas, aerosols, land use-land cover, SST and sea ice, natural and anthropogenic forcings – “hist-1950”; and c) scenario projections from 2015 to 2050 using the SSP585 forcing scenario – “highres-future”. The target resolution for Tier 1 & 2 is set at 25 to 50 km, significantly higher than the typical CMIP6 resolution of 100 km. The data can be accessed from https://hrcm.ceda.ac.uk/research/cmip6-highresmip/. For more detailed information on the experimental design, see Haarsma et al. (2016).

We used data from 8 models from the Tier 2 experiments, as the Tier 1 experiments can be significantly affected by the lack of atmosphere–ocean coupling. Moreover, atmosphere–ocean coupling helps in producing a realistic simulation of the key teleconnections that govern the interannual variability of the monsoon, such as to El Niño (Xavier et al. 2007), and is crucial for any seasonal prediction system (Krishna Kumar et al. 2005). Fully-coupled models, by incorporating dynamic ocean–atmosphere interactions, provide a more accurate representation of ENSO’s impact on South Asia. In contrast, atmosphere-only models with fixed SSTs result in weaker and less realistic ENSO effects, highlighting the necessity of coupled models for reliable teleconnection (Xavier et al. 2007).

The details of the selected models are given in Table 1. We selected high and low resolution versions of models from four model families: CMCC-CM2, HadGEM3-GC31, MPI-ESM1, and EC-Earth3P. This study considered the first ensemble member of all models (i.e., r1i1p1f1). Please note that low-resolution model versions show greater resolution differences than their high-resolution counterparts.**2.4 Rainfall Indices.**

**Table 1:** Details of nine climate/Earth system models from CMIP6 HighResMIP used in the study

S.No	Model	Modelling group	Resolution (latitude x longitude)
1	CMCC-CM2-VHR4	CMCC, Italy	0.23° × 0.31°
2	HadGEM3-GC31-HH	NERC & MOHC, UK	0.24° × 0.35°
3	HadGEM3-GC31-LL	NERC & MOHC, UK	1.2° × 1.2°
4	CMCC-CM2-HR4	CMCC, Italy	0.94° × 1.25°
5	MPI-ESM1-2-HR	MPI, Germany	0.93° × 0.93°
6	MPI-ESM1-2-XR	MPI, Germany	0.46° × 0.46°
7	EC-Earth3P-HR	EC-Earth-Consortium, Europe	0.35° × 0.35°
8	EC-Earth3P	EC-Earth-Consortium, Europe	0.70° × 0.70°

#### Timing of the monsoon

We used the Liebmann et al. (2012) method to determine the onset/withdrawal of the monsoon season which has been previously used by Wainwright et al. (2019) for Africa and a variation thereof by Sperber and Annamalai (2014) for the Indian subcontinent. This accumulation method (method1) uses a timeseries of daily sums of precipitation to calculate the cumulative daily rainfall anomaly *C(d),* given by

$$C\left(d\right)= \sum_{i=1 Jan}^{d}{(Q}_{i}-\overline{Q })$$

where *i* ranges from 1 January to 31 December for each year, ${Q}_{i}$ is the daily rainfall on the *i*th day and $\overline{Q }$ is the annual average daily rainfall. The day of the minimum of *C(d)* marks the beginning of the monsoon season and the day of the maximum marks the retreat (withdrawal hereafter) (Fig. S2). The time period between these two days is the duration of the monsoon season. The results presented in Figs. 3–6 are averaged over the GBM basin. We checked our estimation of the timing of the monsoon using a different fractional accumulation approach (method2) from LinHo and Wang (2002), as discussed in the Supplementary Information. These methods provide dates for each grid point for a given year within our study period, and the average of these dates over the GBM basin is calculated to determine the monsoon timing (onset, withdrawal, and duration) for the entire basin for that year.Both methods focus on local monsoon characteristics, providing insights into the onset and withdrawal of the rainy season within a small region (Bombardi et al. 2020). Moron and Robertson (2014) stated that local onset definitions can effectively capture large-scale interannual monsoon variability, especially with regional synchronization. Additionally, Bombardi et al. (2020) suggested that although statistical methods (such as multivariate regression models with predictors such as ENSO) and dynamical approaches (using climate models) may differ in defining monsoon onset and withdrawal locally, spatial data aggregation could potentially improve predictability by reducing noise and enhancing the regional monsoonal signal. Therefore, defining the monsoon’s timing in a small area could potentially represent the basin-scale timing.

#### Strength of the monsoon

Since the models had output frequencies (3-h and 6-h), we aggregated the rainfall data from the 3-h outputs and reference datasets into 6-h intervals to ensure consistency across all the datasets. We then used the 6-hourly accumulated rainfall from both the models and reference datasets to estimate changes in the strength of monsoon rainfall using three Expert Team on Climate Change Detection and Indices (ETCCDI; Karl et al., 1999) indices:

- Total Rainfall (PRCPTOT): the 6-hourly accumulated rainfall during the monsoon season.
- Annual Maximum Rainfall (Rx6hr): the maximum of 6-hourly accumulated rainfall during the monsoon season.
- 95th percentile of Rainfall (R95p): the 95th quantile of 6-hourly accumulated rainfall for wet days (R >  = 1 mm) during the monsoon season.

To calculate these indices, we normalized the rainfall by dividing the magnitude of rainfall values within a year by the mean annual rainfall for that year. This normalization allows for a fair comparison of the trends in rainfall indices among the different datasets as it attempts to remove the effect of model bias.

## Results & discussion

### Evaluating resolution-related improvements in monsoon rainfall simulation

**Fig. 2:**
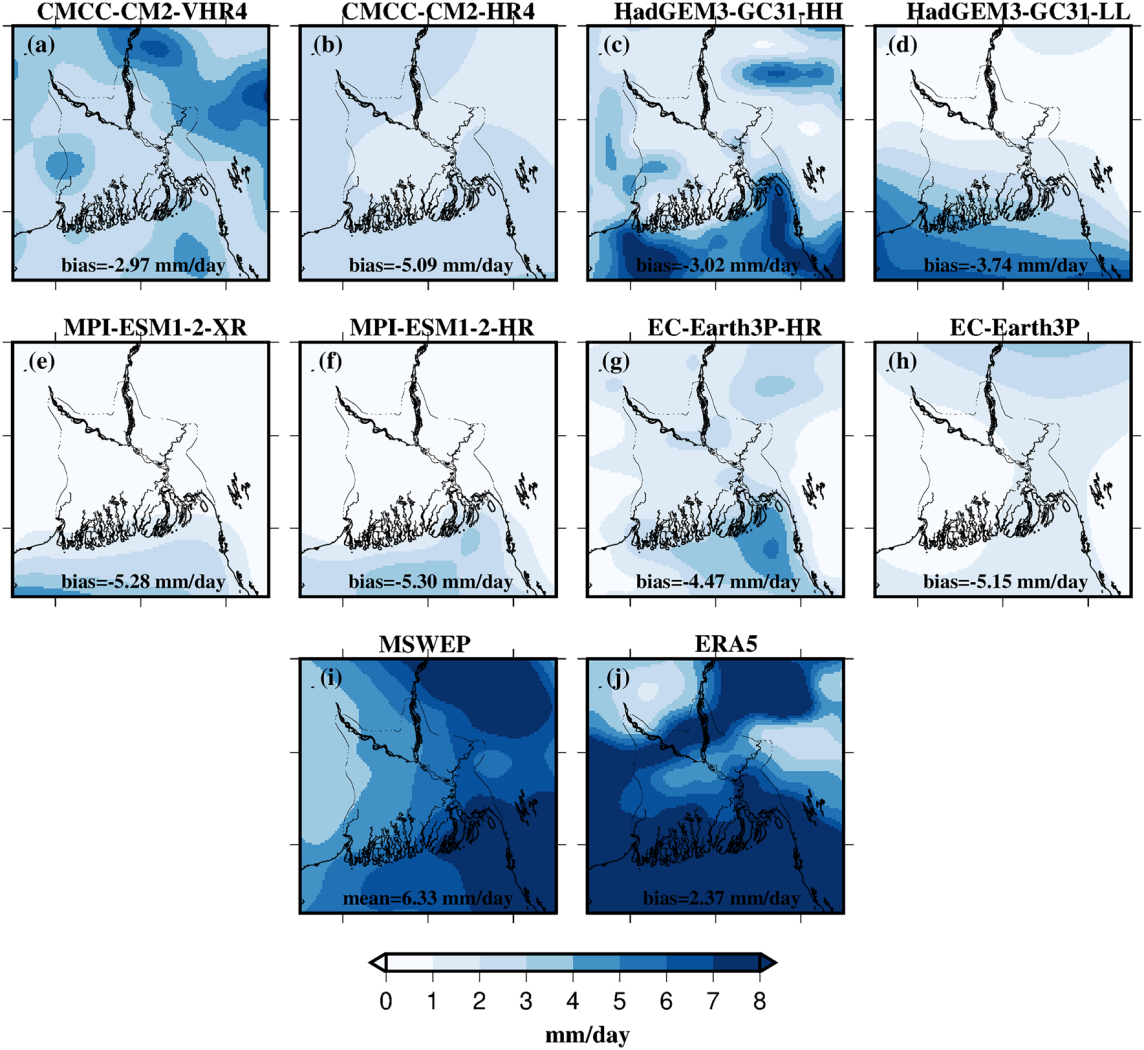
**a, c, e, g** Average annual rainfall (mm/day) for high-resolution models, (b, d, f, h) low-resolution models from four model families, and **i-j** reference datasets (MSWEP & ERA5), for the period 1979–2014. The bias (Bias = R_dataset_ − R_MSWEP_) is calculated as the difference between the average annual rainfall of each dataset and that of MSWEP

We first demonstrate resolution improvement in estimating average annual rainfall against the MSWEP reference dataset by analysing the high- and low-resolution versions of the same models. For example, we compared CMCC-CM2-VHR4 vs. CMCC-CM2-HR4, HadGEM3-GC31-HH vs. HadGEM3-GC31-LL, EC-Earth3P-HR vs. EC-Earth3P, and MPI-ESM1-2-HR vs. MPI-ESM1-2-XR (Fig. 2). The results consistently show that high-resolution models show reduced dry biases (compared to MSWEP) and better simulate average annual rainfall than their low-resolution counterparts. Specifically, Figs. 2c-d highlight the worsened dry bias in the low-resolution version of CMCC-CM2 compared to its high-resolution version. MSWEP shows higher rainfall in the eastern half of the basin, with an average of 6.33 mm/day over the basin (Fig. 2a). However, this east–west contrast is not evident in ERA5, which appears to overestimate average rainfall with a wet bias of 2.37 mm/day compared to MSWEP (Fig. 2b). Overall, while the HighResMIP models fail to capture the spatial pattern of rainfall accurately, they do show a rainfall contrast between the ocean and land. Given the basin’s size and complex topography, the coarser resolution of LR-models may not adequately capture the local processes driving rainfall, highlighting the importance of using finer resolution models as a better alternative to conduct similar studies.

There is a substantial body of literature evaluating global precipitation products against gauge data, but uncertainty remains due to the lack of ground observations, the selection of datasets, and the durations studied. This makes it challenging to evaluate reference datasets before assessing the HighResMIP models. Some confidence comes from global studies using both reanalyses for hydrological applications. For instance, Beck et al. (2017) evaluated 22 precipitation products on a global scale using rain gauges and hydrological modeling, identifying the MSWEP product as one of the top performers. Recently, Xiang et al. (2021) evaluated eight global gridded precipitation products, including MSWEP and ERA5, across 1382 catchments in China, Europe, and North America, finding that MSWEP outperformed ERA5. On the other hand, Baudouin et al. (2020) cross-validated 20 gridded precipitation datatsets in the Indus basin and found precipitation estimates from the ERA5 closest to observations.

### Timing of the monsoon

**Fig. 3:**
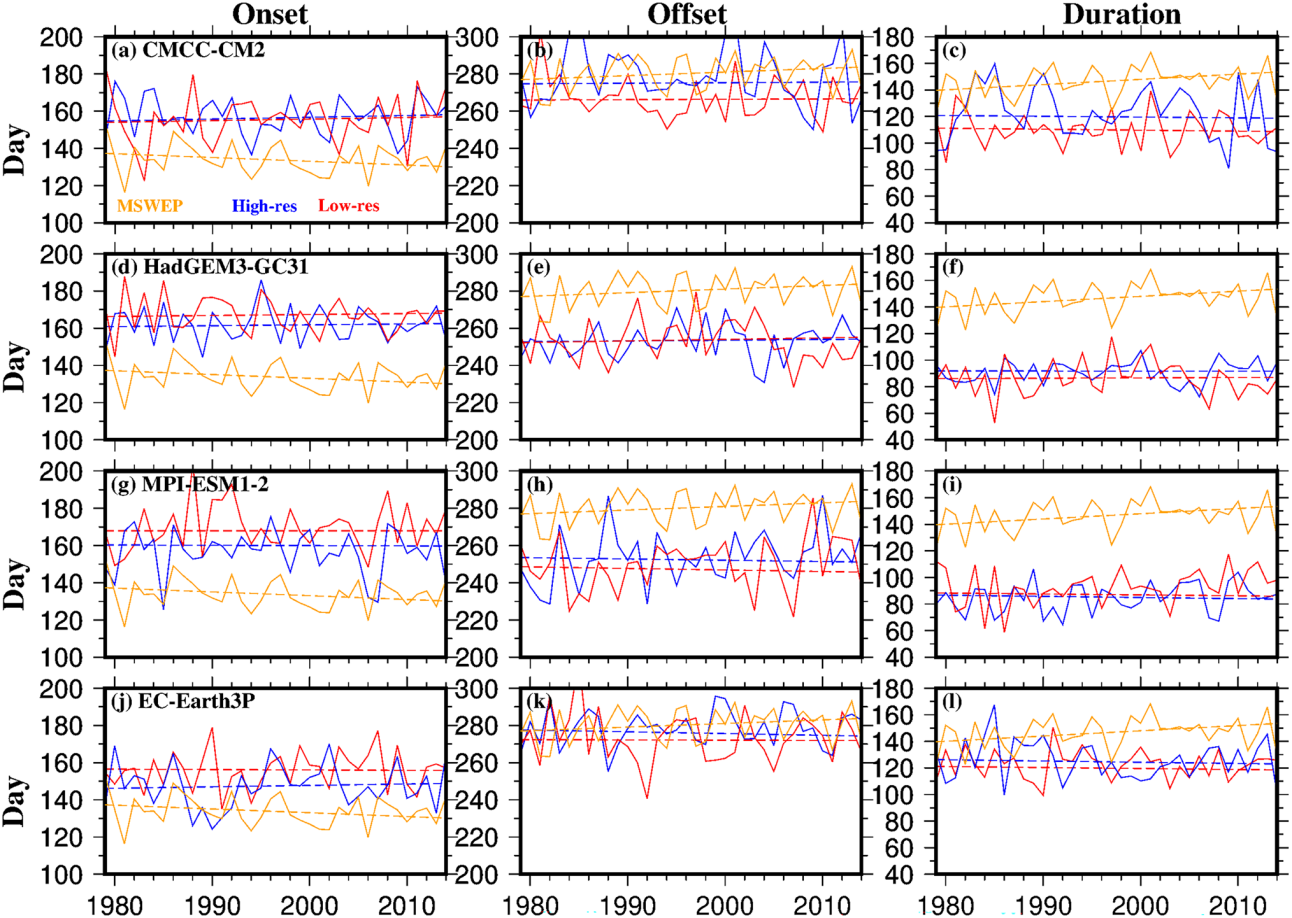
Timing (day of the year) of the monsoon for MSWEP (orange), low-resolution (Low-res; red), and high-resolution (High-res; blue) versions of **a-c** CMCC-CM2, **d-f** HadGEM3-GC31, **g-i** MPI-ESM1-2, and **j-l** EC-Earth3P models for the period 1979–2014. Solid lines represent the area average over the study region, while dashed lines indicate the linear trend (Colour figure online)

We next assessed the simulation of the timing of monsoon rainfall (onset, withdrawal, and duration), averaged across the basin, for both low- and high-resolution versions of the four HighResMIP model families compared to the reference datasets (MSWEP) for the period 1979–2014 (Fig. 3). While we found differences in the monsoon timing between models and MSWEP, these differences were smaller for the high-resolution versions. The low-resolution models showed delayed onset, earlier withdrawal, and shorter monsoon durations compared to the high-resolution versions, which were closer to the MSWEP results.

**Fig. 4:**
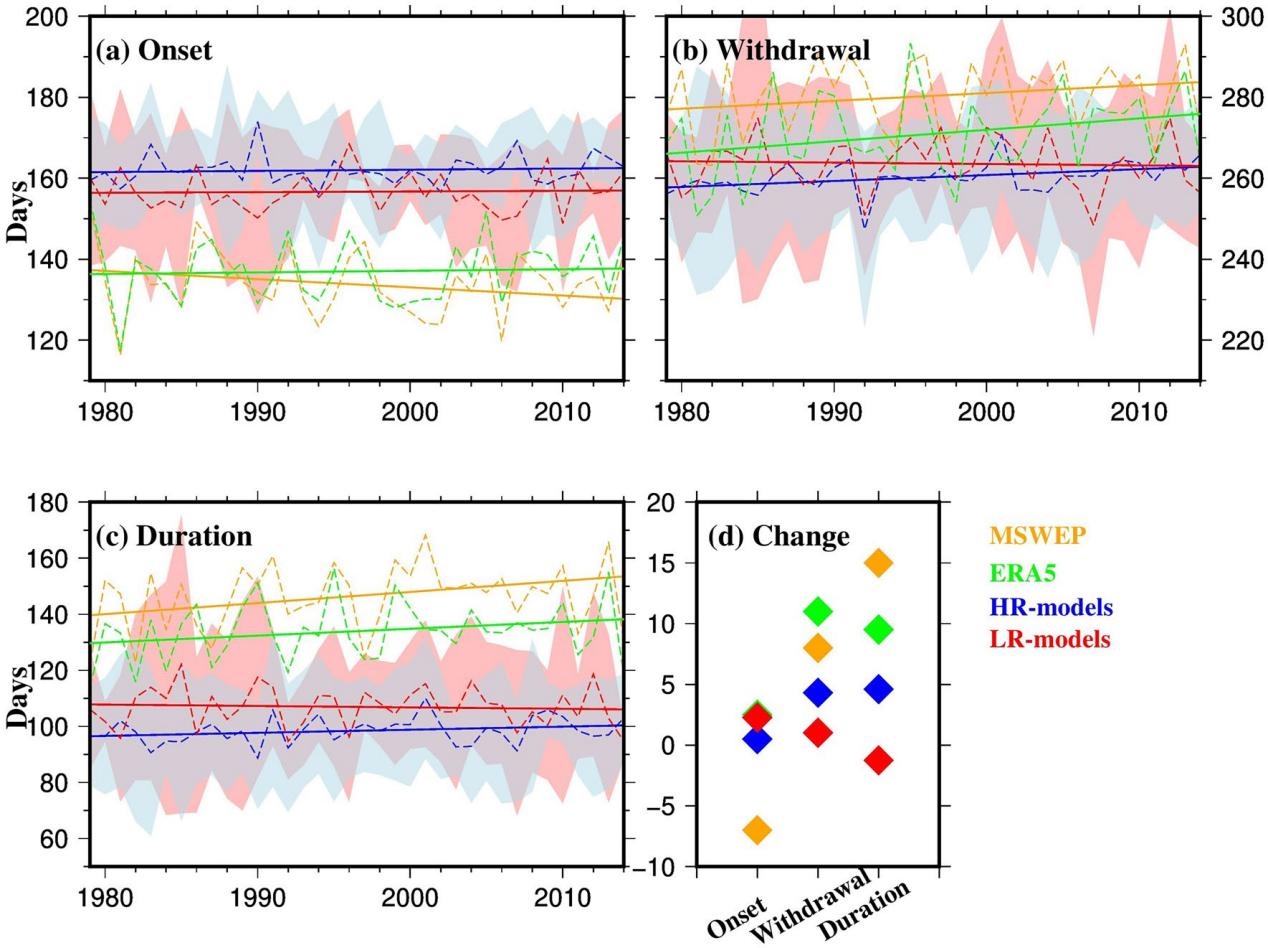
**a-c** Timing (day of the year) and **d** changes (number of days) in the onset, withdrawal, and duration of the monsoon for MSWEP (orange), ERA5 (green), ensemble mean of HR-models (blue), and ensemble mean of LR-models (red) during the historical period 1979–2014. Dashed lines in panels **a-c** represent the year-to-year average over the GBM basin, while solid lines show the linear trend. The ranges in panels **a-c** show the ensemble mean ± one standard deviation for HR-models (light blue) and LR-models (light red). The change in **d** is calculated by multiplying the slope of the linear regression lines by the period duration (i.e., 35 years) (Colour figure online)

We further estimated the multimodel changes in the timing of the monsoon using the ensemble of low- and high-resolution (LR- and HR-models respectively) models from the four model families (Fig. 4). Both reference datasets showed strong agreement (r = 0.84), with a relatively early onset of monsoon rainfall (typically in May), whereas the models showed a later onset, on average in June. ERA5, LR- and HR-models showed a slight positive trend in the onset timing, indicating a delay of up to 3 days (calculated by multiplying the regression slope of the onset with the duration), while MSWEP showed an earlier onset by up to 7 days (Fig. 4a, d). The variability in onset timing was greater for the LR-models compared to the HR-models, which may reflect differences in model resolution.

For monsoon withdrawal, ERA5 and LR-models showed a late withdrawal, with the reference datasets indicating a shift towards the end of September, while the HR-models showed an average withdrawal much earlier in August (Fig. 4b). All datasets except LR-models, however, showed a trend towards a delayed withdrawal by the end of 2014. The increase in monsoon duration was observed in both reference datasets, with MSWEP showing the largest increase of up to 15 days, followed by ERA5 (10 days) and HR-models (5 days), However, LR-models showed a decrease of 1 day in the duration of monsoon by 2014. This suggests an extension of the monsoon season across all datasets except LR-models, with MSWEP reflecting the largest change (Fig. 4c, d). Method2, used for validating the timing of onset and withdrawal, provided similar results for onset but showed a consistent delay in withdrawal compared to Method1 across all models (Fig. S2). The differences might be due to the low threshold used in Method2, which may have been influenced by winter rainfall biases, resulting in delays in the withdrawal estimation.

**Fig. 5:**
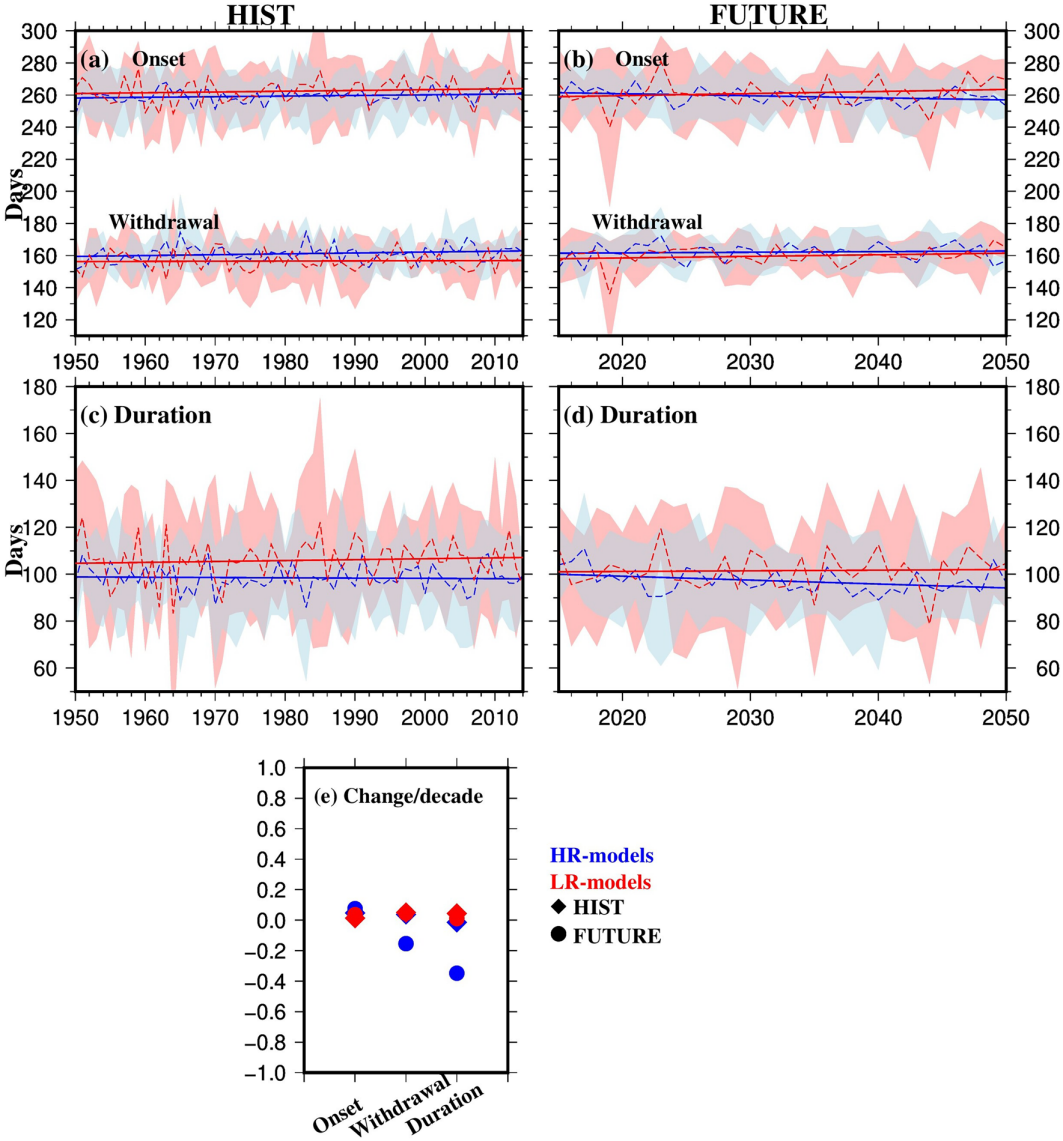
Timing (day of the year) in the onset and withdrawal of the monsoon during the **a** HIST period (1950–2014), **b** FUTURE period (2015–2050), for ensemble mean of HR-models (blue) and ensemble mean of LR-models (red), and **c-d** duration of the monsoon season during the HIST and FUTURE periods respectively, and **e** change per decade (number of days) of ensemble mean of onset, withdrawal and duration of the monsoon for the HIST (diamonds) and FUTURE (circles). Dashed lines in panels **a-d** represent the year-to-year average over the GBM basin, while solid lines show the linear trend. The ranges in panels **a-d** show the ensemble mean ± one standard deviation for HR-models (light blue) and LR-models (light red) (Colour figure online)

Next, we examined long-term trends in monsoon timing by comparing the HIST (1950–2014) and FUTURE (2015–2050) periods, using high-resolution future simulations (2015–2050) (Fig. 5). For both periods, we observed a delay in monsoon onset for HR-models, with more pronounced trends in the FUTURE period. Specifically, while HR-models show a slight delay in onset during the HIST period, they exhibit a slight early onset by the end of the FUTURE period. Additionally, the monsoon duration is projected to decrease in the FUTURE period, with a more decline in HR-models (-1.1%/decade) compared to the HIST period (-0.38%/decade). This suggests that the monsoon season may shorten in the future, particularly in higher-resolution models (Fig. 5c-e). Table 2 summarizes the results on the observed and projected changes in the monsoon timing, as presented in Figs. 4 and 5.

**Table 2:** Change (number of days) per decade in the timing of monsoon

Period	Dataset	Onset	Withdrawal	Duration
1979–2014	MSWEP	− 1.94	2.22	4.16
	ERA5	0.83	3.05	2.23
	HR-models	0.21	1.12	1.35
	LR-models	0.79	0.25	− 0.54
FUTURE (2015–2050)	HR-models	0.11	− 0.19	− 0.32
	LR-models	0.05	0.08	0.03
HIST (1950–2014)	HR-models	0.02	0.03	-0.01
	LR-models	− 0.01	0.03	0.03

The uncertainty in results from coupled models arises from their limitations in representing various aspects of the monsoon, particularly due to inaccuracies in representing physical processes like convection and SSTs, which are common biases in these models (Bollasina and Ming, 2013; Sperber et al. 2013). Coupled CMIP-class models often have cold biases in the Arabian Sea, which leads to reduced evaporation and moisture fluxes that feed into the monsoon during summer (Levine et al., 2012, 2013). As a result, these cold SST biases significantly contribute to the delayed monsoon onset in coupled models compared to reference datasets (Levine et al. 2013; Menon et al. 2018). The limitation in simulating accurate SST can be partly addressed by increasing the models’ horizontal resolution. For instance, Bhattacharya et al. (2022) found that CMIP6 high-resolution models produce more accurate Arabian SSTs with a reduced cold bias compared to lower-resolution models.

Our results, which show trends in the observed timing of monsoon rainfall based on the HR-model ensemble, align with the findings of Montes et al. (2021). The observed delay in the onset and withdrawal of the monsoon over the GBM basin, along with the extended monsoon duration, is attributed to a complex set of factors including anthropogenic climate change, land-use changes, and atmospheric pollution (Dong et al., 2016; Montes et al. 2021; Sun et al. 2023, 2017). Conversely, HR-model projections suggest an earlier onset and withdrawal, leading to a reduction in monsoon duration. Studies indicate that global warming may reduce the upper tropospheric meridional temperature contrast due to enhanced tropical diabatic heating, which could partially offset the enhanced lower tropospheric contrast, potentially weakening the monsoon and delaying its onset (Sun et al. 2010). Additionally, global warming can induce shifts in tropical circulation patterns, influencing monsoon dynamics and further contributing to a delayed onset (Vecchi and Soden 2007; Zhang et al. 2013). The IPCC AR6 (Chapters 8 and 10) suggests medium confidence in the projected weakening of the South Asian monsoon circulation, which is expected to alter the spatial distribution and timing of rainfall, with potential delays in onset and shifts in withdrawal patterns (Douville et al., 2021; Doblas-Reyes et al. 2021).

Previous research has increasingly focused on the physical and dynamical processes underlying the trends and variability of the South Asian monsoon. Roxy (2014) highlight the significant role of changes in sea surface temperatures (SSTs) in modulating the onset, intensity, and duration of the monsoon, in conjunction with atmospheric dynamics. Furthermore, the interaction between oceanic and atmospheric phenomena, such as the Indian Ocean Dipole (IOD) and El Niño-Southern Oscillation (ENSO), plays a crucial role in shaping monsoon behavior, with studies showing significant teleconnections between these phenomena and monsoon characteristics (Cherchi et al. 2021). These studies underscore the importance of understanding how changes in oceanic conditions, such as SST anomalies, and atmospheric circulation patterns are driving recent shifts in monsoon characteristics. A more detailed understanding of these processes is critical for improving projections of future monsoon behavior and enhancing climate resilience in the South Asian region.

### Strength of the monsoon

**Fig. 6:**
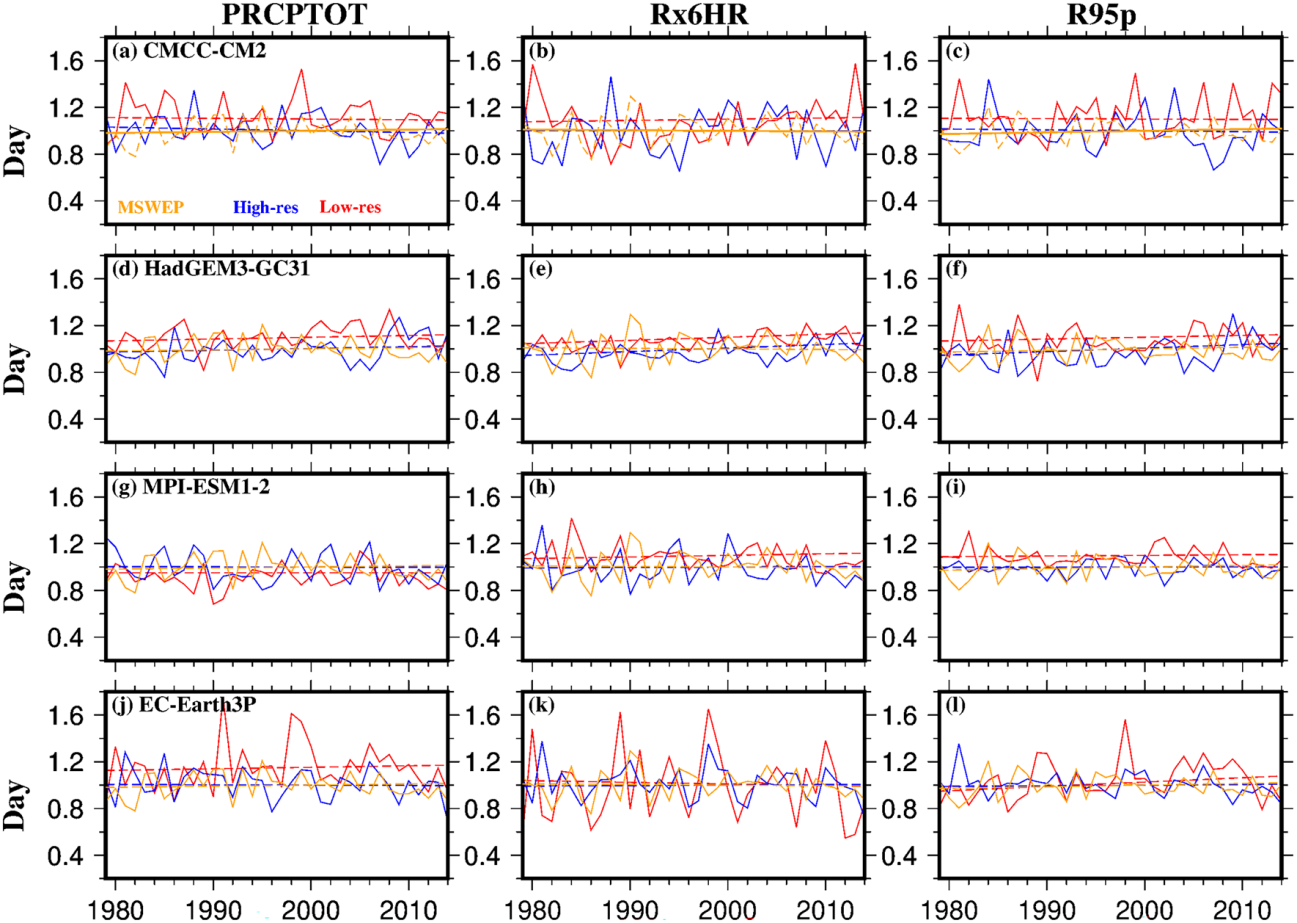
Rainfall indices (Total rainfall, PRCPTOT; annual maximum rainfall, Rx6HR; 95th percentile of rainfall (for wet days), R95p) of the monsoon (June–September) for MSWEP (orange), low-resolution (Low-res; red), and high-resolution (High-res; blue) versions of **a-c** CMCC-CM2, **d-f** HadGEM3-GC31, **g-i** MPI-ESM1-2, and **j-l** EC-Earth3P models for the period 1979–2014. Solid lines represent the area average over the study region, while dashed lines indicate the linear trend (Colour figure online)

We compared the strength of the monsoon, focusing on total (PRCPTOT) and extreme (Rx6HR & R95p) rainfall indices, against MSWEP for both LR- and HR-models for the period 1979–2014 (Fig. 6). This analysis highlights the importance of using high-resolution model versions, as they show better agreement with MSWEP results. In contrast, the low-resolution versions tend to overestimate the strength of the monsoon in observations. However, the difference in the trends of these indices was not found to be statistically significant (p < 0.05).

**Fig. 7:**
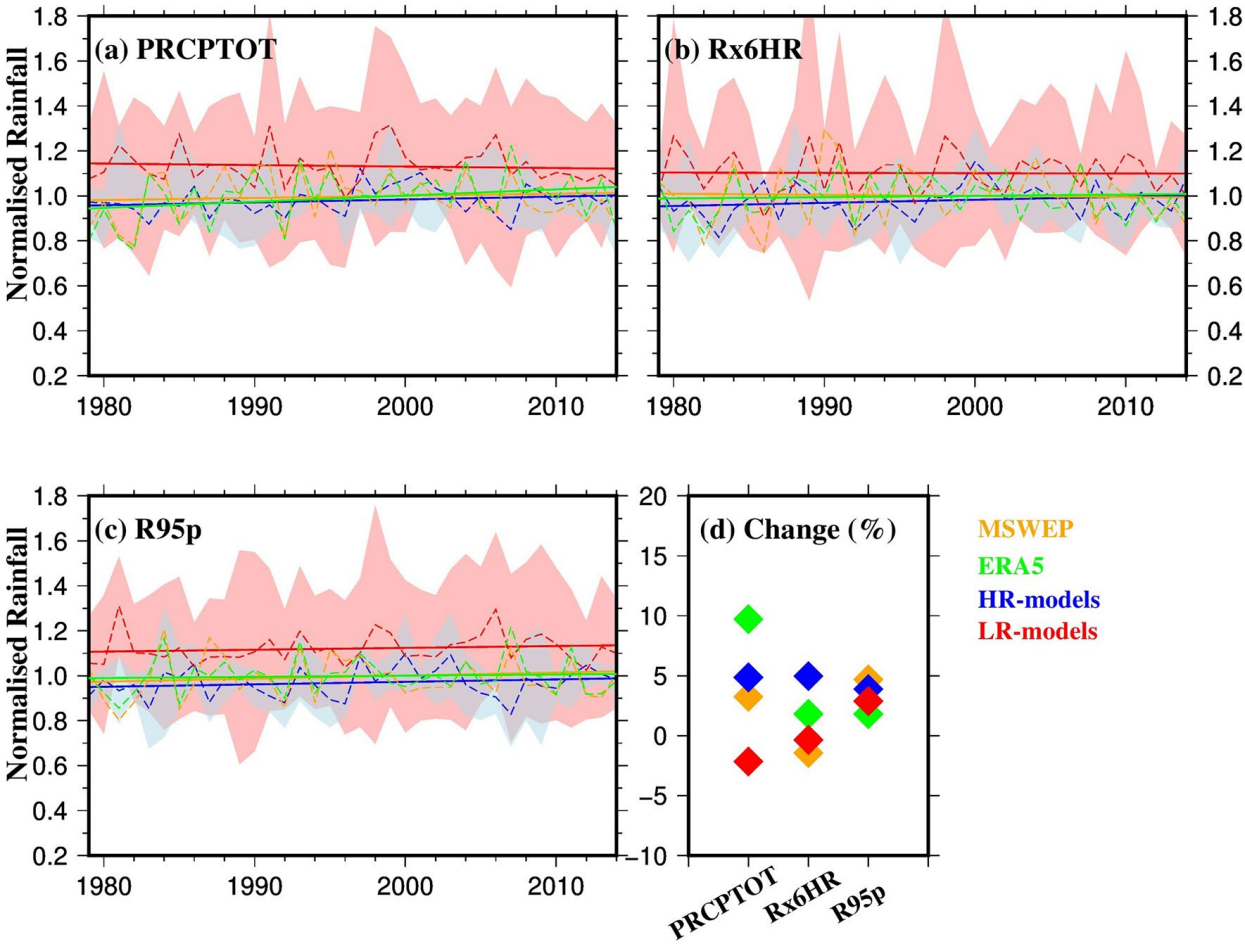
**a-c** Rainfall indices (Total rainfall, PRCPTOT; annual maximum rainfall, Rx6HR; 95th percentile of rainfall (for wet days), R95p) of the monsoon (June–September) for MSWEP (orange), ERA5 (green), ensemble mean of HR-models (blue), and ensemble mean of LR-models (red) during the historical period 1979–2014 and **d** change (in percentage; regression slope × duration) in the rainfall indices for the period 1979–2014. Dashed lines in panels **a-c** represent the year-to-year average over the GBM basin, while solid lines show the linear trend. The ranges in panels **a-c** show the ensemble mean ± one standard deviation for HR-models (light blue) and LR-models (light red). The normalised rainfall for each model was calculated by dividing the magnitude of the rainfall within a year by its mean annual rainfall (Colour figure online)

We next analyzed changes in the strength of monsoon rainfall using the reference datasets (MSWEP & ERA5) and ensembles of HR- and LR-models for the historical period 1979–2014 (Fig. 7). Due to a large bias in the average annual rainfall across the models (Fig. 2), we calculated trends in normalized rainfall averaged over the GBM basin to ensure a fair comparison. Our findings show a relatively similar linear trend in the change of PRCPTOT between HR-models (5%) and the reference datasets (up to 10%) during the historic period (Fig. 7a, d). In contrast, LR-models show a decline (2%) in PRCPTOT, capturing higher annual variability (Fig. 7). For the Rx6HR index, LR-models and MSWEP show a decrease (~ 2%), while HR-models and ERA5 show increases of 5% and 2% respectively (Fig. 7b, d). Notably, all datasets show an increasing trend in R95p (up to 5%) during the historic period.

**Fig. 8:**
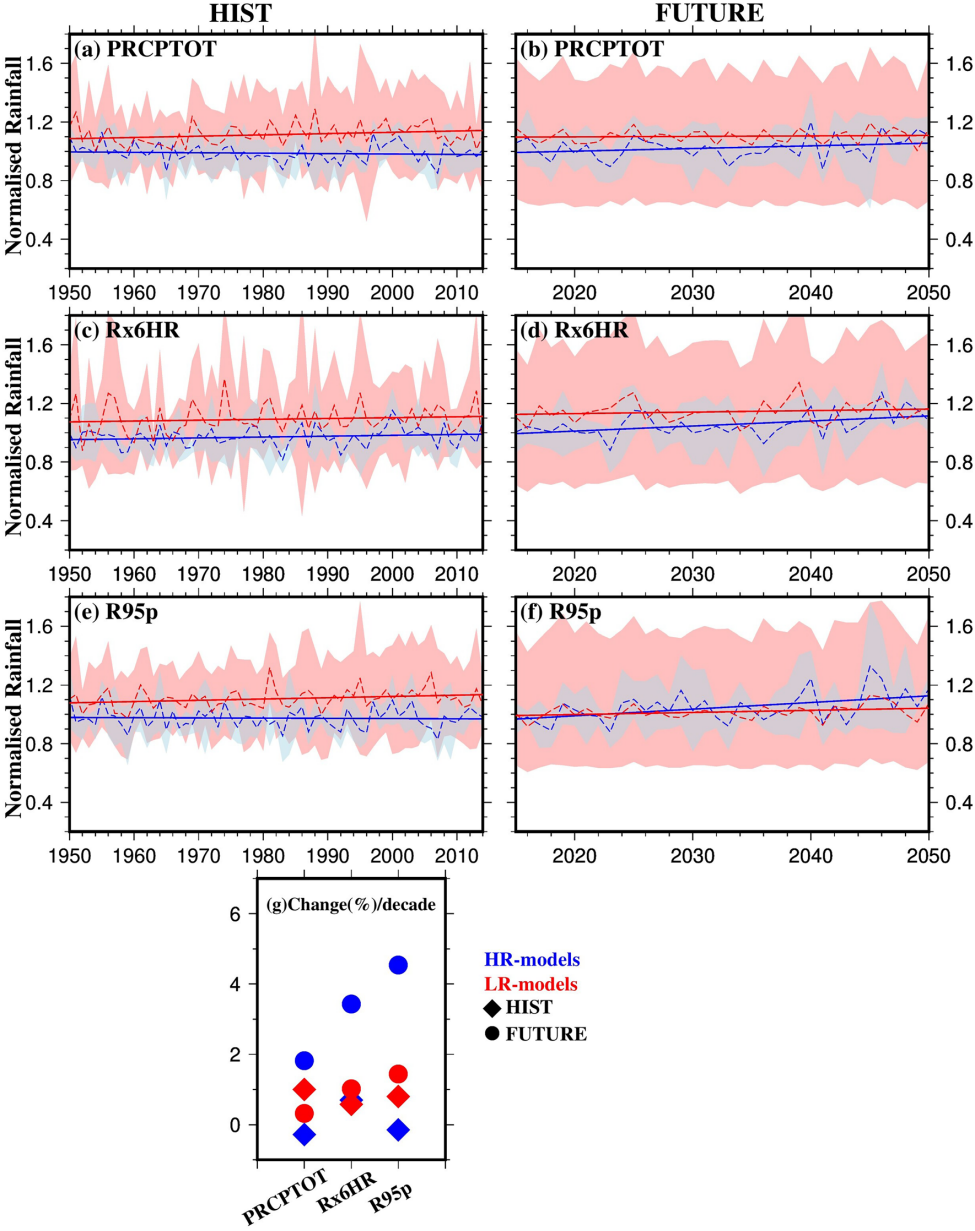
Rainfall indices (Total rainfall, PRCPTOT; annual maximum rainfall, Rx6HR; 95th percentile of rainfall (for wet days), R95p) of the monsoon (June–September) during the **a, c, e** HIST period (1950–2014), **b, d, f** FUTURE period (2015–2050) respectively, for ensemble mean of HR-models (blue) and ensemble mean of LR-models (red), and **g** change per decade (in percentage; regression slope × duration) in the rainfall indices of monsoon for the HIST (diamonds) and FUTURE (circles). Dashed lines in panels **a-f** represent the year-to-year average over the GBM basin, while solid lines show the linear trend. The ranges in panels **a-f** show the ensemble mean ± one standard deviation for HR-models (light blue) and LR-models (light red). The normalised rainfall for each model was calculated by dividing the magnitude of the rainfall within a year by its mean annual rainfall (Colour figure online)

We also assessed the projected changes in rainfall indices between the HIST and FUTURE periods (Fig. 8). All models show increasing trends for all indices in the FUTURE period. Specifically, HR-models show an average increase of ~ 1.8%, ~ 3.8%, and ~ 4.8% per decade for PRCPTOT, Rx6HR, and R95p, respectively, in the FUTURE period. LR-models show lower increases with a wider range (mean ± standard deviation) for the FUTURE period. Our results indicate a larger projected increase in extreme monsoon rainfall compared to total monsoon rainfall, particularly in the more realistic HR-models. Table 3 summarizes the results on monsoon strength, as presented in Figs. 7 and 8. Both tables (2 and 3) are organized to align with the research questions stated in the introduction, making it easier for readers to assess the performance of HighResMIP models, identify observed and projected changes, and evaluate the benefits of increased resolution.

**Table 3:** Regression slopes per decade (in percentage) in the strength of monsoon

Period	Dataset	PRCPTOT	Rx6HR	R95p
1979–2014	MSWEP	0.91	− 0.43	1.31
	ERA5	2.78	0.52	0.56
	HR-models	1.42	1.44	1.22
	LR-models	− 0.85	− 0.28	0.98
FUTURE (2015–2050)	LR-models	0..98	1.25	1.32
	HR-models	1.34	3.67	5.56
HIST (1950–2014)	LR-models	0.23	0.32	0.39
	HR-models	0.02	0.03	− 0.008

It is important to note that the larger variability observed among the LR models may, in part, be a consequence of the greater disparity in their native horizontal resolutions. This variation in resolution can significantly affect how well key physical processes—such as precipitation, storm structure, and cyclone dynamics—are represented across models. For instance, coarser-resolution models may struggle to capture small-scale features of storm systems, leading to greater differences in their simulated outputs. In contrast, the HR models in our ensemble have more similar and finer horizontal resolutions, which allows for a more consistent and detailed representation of these processes. This likely explains the smaller inter-model spread seen in the HR ensemble, particularly in Figs. 4, 5, 7, and 8.

The difference in trends simulated by the HR-models and LR-models, as noted by Bador et al. (2020), highlight the significant increase in rainfall extremes over the tropics, which is underestimated by the LR-models. Our findings are consistent with previous studies focussing on GBM basin regions (Bhattacharjee et al. 2023; Kamruzzaman et al. 2023; Das et al. 2022) which project increased monsoon rainfall over Bangladesh and eastern India under all RCP scenarios. For example, Almazroui et al. (2020) reported a rise in monsoon rainfall by 7.5–36.9% (for SSP-8.5) by the end of the twenty-first century across Bangladesh, which is a key part of the GBM delta.

There has been a lot of discussion about trends in monsoon rainfall, with recent studies showing mixed results. Over the past century, rainfall trends in South Asia have shown significant interannual and spatial variability, with an overall weakening of the monsoon since the 1950s (Kulkarni et al., 2012; Jamshadali et al. 2021). While average rainfall might not show a significant increasing trend in the observed record, the frequency and intensity of heavy rainfall events have risen (Ali et al. 2019; Goswami et al. 2006; Shahid 2011). Future projections from CMIP5 models also suggest that heavy rainfall events will increase due to higher moisture content in the atmosphere (Sooraj et al. 2015). The IPCC AR6 (Chapter 10) points out that global warming will likely lead to more intense and frequent rainfall events in monsoon regions, where extreme events have already become more common (Doblas-Reyes et al. 2021). This is largely due to increased atmospheric moisture from warming, which is expected to drive more intense monsoon rainfall. Additionally, Chapter 8 of the IPCC AR6 (Douville et al., 2021) expresses high confidence that rainfall extremes will increase in the Indian monsoon region due to global warming.

Previous studies have debated whether increasing horizontal resolution, such as in the HighResMIP models, improves model performance. For instance, Xin et al. (2021) found that higher-resolution models (30–50 km) performed better than lower-resolution models (70–140 km) in capturing rainfall patterns over northwest and southwest China. This improvement was mainly due to the higher-resolution models’ ability to better represent topographical rainfall and local vertical circulation in complex terrain. Moreover, Liang et al. (2022) showed that HighResMIP models with higher horizontal and vertical resolutions performed better in simulating total rainfall, capturing the observed annual cycles, spatial rainfall patterns, and the link between rainfall and monsoon intensity in peninsular Malaysia from 2001 to 2014, compared to coarser-resolution models. In contrast, Avila-Diaz et al. (2023) found no strong correlation between increased resolution and improved performance in simulating rainfall extremes across Latin America and the Caribbean.

Recent studies such as Sorland et al. (2016) and Bohlinger et al. (2019) have also used the Weather Research and Forecasting (WRF) model with resolutions as fine as 4 km to better represent local topography and fine-scale processes, enhancing monsoon rainfall simulations. However, these models come with limitations, including high computational costs, biases in large-scale dynamics, and reliance on parameterizations for unresolved processes like convection and cloud microphysics (Bohlinger et al. 2019). Validation is further constrained by the lack of high-resolution observational data in many regions. While these models are better in capturing spatial rainfall patterns and extremes, they often struggle with temporal trends and large-scale monsoon drivers, sometimes overestimating extreme events (Chawla et al. 2018). Therefore, it is crucial to choose an appropriate resolution of models to study monsoon characteristics. We emphasise that the HR-models within the HighResMIP framework offer some improvement in reliability in projecting potential future changes in rainfall under a warming climate, although their performance may vary based on the specific study region and phenomena of interest.

## Conclusion

This study presents a novel framework for evaluating the impact of model resolution on the simulation of South Asian monsoon rainfall, demonstrating that high-resolution models provide more accurate representations of monsoon timing, intensity, and duration compared to their low-resolution counterparts. High-resolution models consistently outperform low-resolution versions in simulating key aspects of monsoon behavior, including average annual rainfall, monsoon timing, and strength, with better alignment to the MSWEP reference dataset. While differences between model resolutions were evident, the trends in simulation outcomes were not statistically significant (p > 0.05).

We analysed changes in the timing and strength of the monsoon in the GBM basin using reference datasets (MSWEP & ERA5) alongside ensembles of high- and low-resolution models from the CMIP6 HighResMIP. Our results revealed significant shifts in both monsoon timing and intensity from 1979 to 2014. All datasets indicated a delay in monsoon withdrawal by the end of 2014, with delays observed in ERA5 (up to 12 days) and MSWEP (up to 8 days). Monsoon duration increased by up to 15 days for MSWEP, 10 days for ERA5, and 4 days for high-resolution models, while low-resolution models showed a decrease of 2 days in monsoon duration. Notably, high-resolution models showed a delayed onset of the monsoon compared to the reference datasets, with a shift of 1 day toward later onset and a 4-day delay in withdrawal, resulting in an overall increase in monsoon duration. In contrast, low-resolution models exhibited more pronounced delays in both onset and withdrawal. Projections for the future (2015–2050) suggest a further delay in monsoon onset, with more notable delays in the future period compared to the historical period. This shift is expected to lead to a reduction in overall monsoon duration in the future.

In terms of monsoon strength, we found that from 1979–2014, both high-resolution models (HR-models) and the reference datasets showed similar trends in total precipitation (PRCPTOT), with HR-models displaying a 5–6% increase compared to up to 10% in the reference datasets. Low-resolution models (LR-models) showed a decline of 2% in PRCPTOT, with higher annual variability. For the Rx6HR index, both LR-models and MSWEP observations displayed a decline of about 2%, while HR-models and ERA5 reanalysis showed increases of 5–10% and 2%, respectively. Additionally, all datasets indicated an increasing trend in extreme rainfall events (R95p), with up to a 5% rise during the period from 1979–2014. Looking ahead to the future (2015–2050), all models project increases in strength indices, with HR-models showing greater increases in PRCPTOT (~ 2%/decade), Rx6HR (~ 3.6%/decade), and R95 (~ 4.5%/decade) compared to LR-models. Notably, LR-models, which had higher increases than HR-models in the historical period, are expected to show smaller increases and greater variability in the future.

The projected increase in monsoon rainfall can be primarily attributed to the intensified thermodynamic conditions driven by global warming (Meehl et al., 2003). The Clasius-Clapeyron relationship indicate that for every 1 °C increase in temperature, the atmosphere’s moisture-holding capacity increases by approximately 7%, which is evident in several observational studies, and can be higher than 7% for sub-daily extreme rainfall (Ali et al. 2021b; 2022). This enhanced moisture retention, driven by global warming, significantly contributes to a higher long-term rainfall rate, especially during intense rainfall events (Ali et al., 2017). Additionally, studies (IPCC AR6; Shahi et al. 2023) utilising CMIP6 models across India have found a robust correlation between global warming and the projected increase in the frequency and intensity of extreme rainfall events. Specifically, in the GBM basin, the Bay of Bengal acts as the primary moisture source for monsoon-related thunderstorms. Rising SSTs in the Bay of Bengal are expected to strengthen the atmospheric circulation, potentially resulting in stronger and more persistent winds that could further amplify rainfall patterns in Bangladesh (Bhattacharjee et al. 2023). Although the increase in total monsoon rainfall may benefit crop irrigation, the projected increase in extreme rainfall poses significant risks to the GBM delta, potentially making it more vulnerable to severe flash flooding, leading to flood hazards, crop damage, and soil erosion.

The local-scale definitions used to define the monsoon timing in our study could be further refined by considering factors such as rainfall event duration and dry periods during the monsoon season to improve accuracy. Additionally, while our study focuses on rainfall characteristics, recent work by Li et al. (2014) expands on the study of monsoon timing by considering a broader range of indices, including those based on meridional wind (e.g., Monsoon Hadley Circulation Index), atmospheric temperature (e.g., Tropospheric Temperature Gradient), outgoing longwave radiation (e.g., Convection Index), and the hydrological cycle. These indices provide valuable insights into the mechanisms governing monsoon onset and progression, offering an opportunity for further research to integrate multiple factors for a comprehensive understanding of the monsoon's timing and strength across different models and future projections. Such multi-dimensional approaches could significantly improve the accuracy of climate projections, particularly in vulnerable regions like the GBM basin, where the impacts of monsoon variability are felt most acutely.

Overall, this study emphasizes the importance of model resolution in accurately simulating monsoon characteristics, particularly in South Asia, where the monsoon is crucial for water and agriculture. By comparing high- and low-resolution models, we gain insights into how resolution affects monsoon timing and strength, helping to refine future models and improve climate projections. These findings also provide a foundation for future research on model resolution's role in climate simulations and will inform adaptation strategies to manage monsoon-related risks.

## Supplementary Information

Below is the link to the electronic supplementary material.

Supplementary file1 (DOCX 566 KB)

## Data Availability

This article draws on data that will be made available via Newcastle University’s Research Repository (https://data.ncl.ac.uk/). The data will be available from March 2025 onwards, as part of the data generated by the GCRF UKRI-funded Living Deltas Hub (2019–2024) under Grant Reference NE/S008926/1. https://doi.org/10.25405/data.ncl.c.6288033.v1
