# Supplementary material for: High-resolution climate models improve simulation of monsoon rainfall changes in the Ganga–Brahmaputra-Meghna basin
Source: Clim Dyn. 2025 Jun 6;63(6):246. doi: 10.1007/s00382-025-07716-6 (PMC12144052; doi:10.1007/s00382-025-07716-6)
Supplement: Supplementary file 1 — Supplementary file1 (DOCX 566 KB) [file 382_2025_7716_MOESM1_ESM.docx]

**High-resolution climate models improve simulation of monsoon rainfall changes in the Ganga-Brahmaputra-Meghna basin**

Haider Ali^1, 2^, Hayley J. Fowler^1, 2^ & Andrew G. Turner^3,4^

1. School of Engineering, Newcastle University, Newcastle upon Tyne, UK

2. Tyndall Centre for Climate Change Research, Newcastle University, Newcastle upon Tyne, UK

3. National Centre for Atmospheric Science, University of Reading, Reading, UK

4. Department of Meteorology, University of Reading, Reading, UK

**Supplemental Information**

**Methodology:**

**LinHo & Wang (2002) method:**

The methodology involves smoothing pentad time series with a five pentad running mean and then removing December to January mean (R_mean_) rainfall to calculate the relative rainfall rate. This method (method2) reduces high-frequency fluctuations while preserving climatological intraseasonal oscillation (LinHo and Wang 2002). For each grid, the monsoon is identified when the relative rainfall rate exceed Rmean from May to September. The onset is the first pentad meeting this threshold, peak and monsoon withdrawal is when the rate falls below R_mean_. Monsoon duration is calculated as: (decay pentad) minus (onset pentad) (Fig. S2b).

**
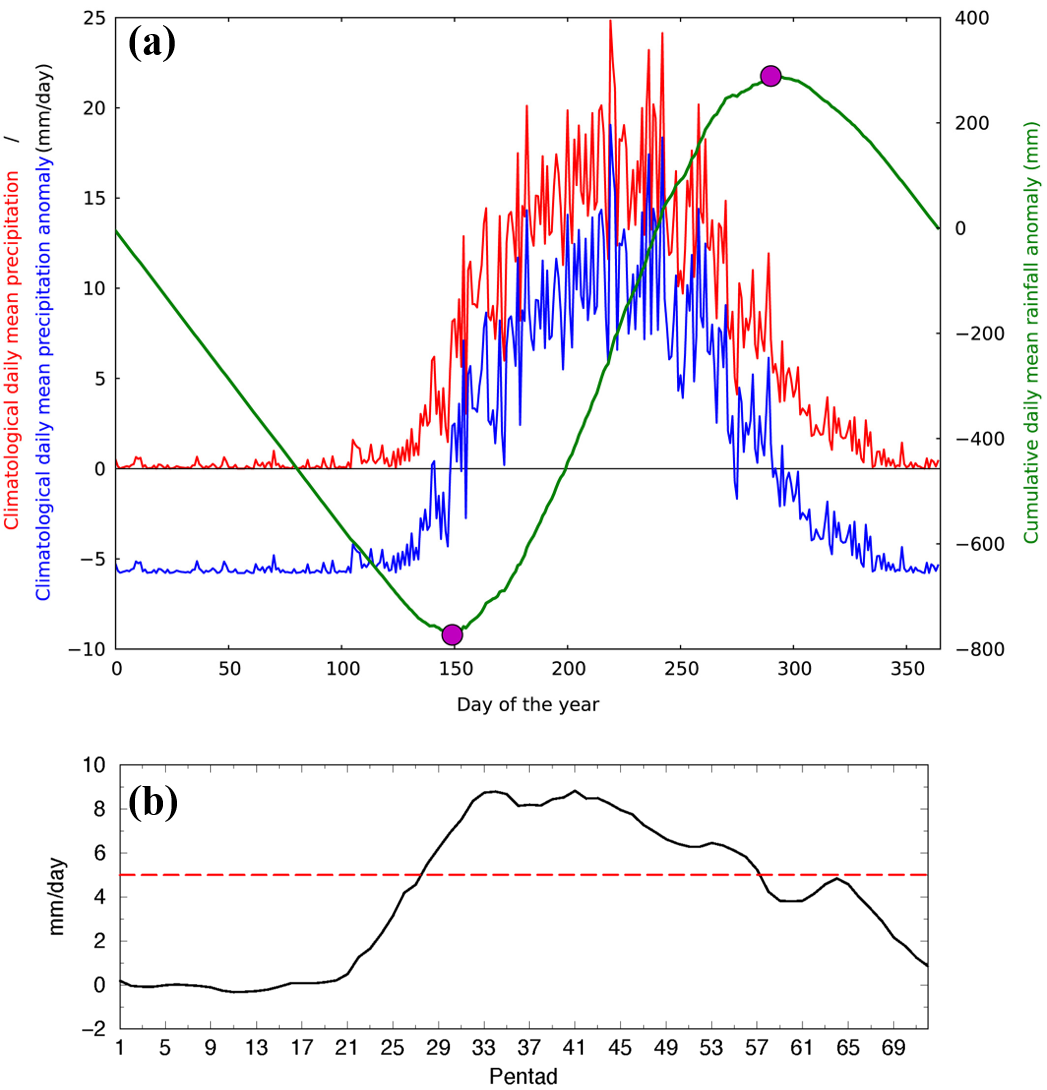
**

Figure S1 (a): Taken from Dunning et al. (2016). Depiction of daily mean rainfall (red), daily mean rainfall anomaly (blue), and cumulative daily mean rainfall anomaly (green). Magenta dots indicate the monsoon season duration, and (b): Taken from Sperber et al. (2013). Illustration of relative rainfall rate using pentads. The black line represents smoothed pentad rainfall, with the red line indicating the mean December to January rainfall used as a threshold for calculating relative rainfall rate.


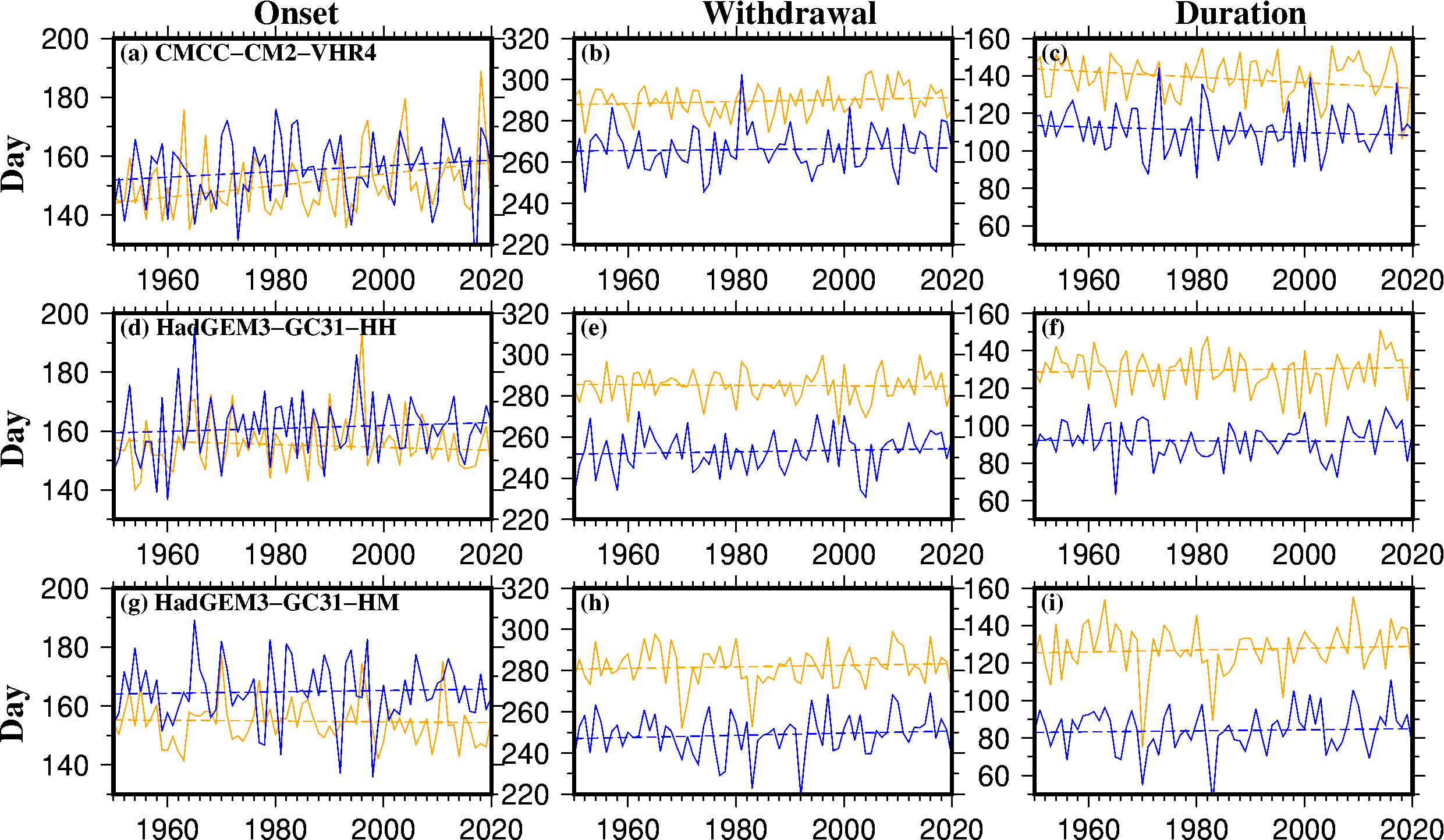


Figure S2. Timing of the monsoon for the (a-c) CMCC-CM2-VHR4 model, (d-f) HadGEM3-GC31-HH model and (g-i) HadGEM3-GC31-HM model using the Liebmann et al. (2012) method (blue; method1) and LinHo and Wang (2002) method (orange; method2) respectively during the period 1950-2020. The solid lines are the areal average over the study region while the dashed lines are the linear trend.

**References**

Dunning, C. M., Black, E. C., & Allan, R. P. (2016). The onset and cessation of seasonal rainfall over Africa. *Journal of Geophysical Research: Atmospheres*, *121*(19), 11-405.

Sperber, K. R., Annamalai, H., Kang, I. S., Kitoh, A., Moise, A., Turner, A., ... & Zhou, T. (2013). The Asian summer monsoon: an intercomparison of CMIP5 vs. CMIP3 simulations of the late 20th century. *Climate dynamics*, *41*, 2711-2744.
